# The outcome of surgically treated traumatic unstable pelvic fractures by open reduction and internal fixation

**DOI:** 10.5249/jivr.v5i2.138

**Published:** 2013-06

**Authors:** Keykhosro Mardanpour, Mahtab Rahbar

**Affiliations:** ^a^Faculty Member of Kermanshah University of Medical Sciences, Kermanshah, Iran.

**Keywords:** Trauma, Outcome, Unstable Pelvic fracture, Internal fixation, Open reduction

## Abstract

**Background:**

This study was performed to evaluate functional and radiological results of pelvic ring fractures treatment by open reduction and internal fixation.

**Methods:**

Thirty eight patients with unstable pelvic fractures, treated from 2002 to 2008 were retrospectively reviewed. The mean patients’ age was 37 years (range 20 to 67). Twenty six patients were men (4patients with type B and 22 patients with type C fracture) and 12 women (7 patients with type B and 5 patients with type C fracture). The commonest cause was a road traffic accident (N=37, about 97%). Internal fixation was done by plaque with ilioinguinal and kocher-langenbeek approaches for anterior, posterior pelvic wall and acetabulum fracture respectively. Quality of reduction was graded according to Majeed score system.

**Results:**

There were 11 type-C and 27 type-B pelvic fractures according to Tile’s classification. Thirty six patients sustained additional injuries. The commonest additional injury was lower extremity fracture. The mean follow-up was 45.6 months (range 16 to 84 months).The functional outcome was excellent in 66%, good in 15%, fair in 11% and poor in 7% of the patients with type B pelvic fractures and functional outcome was excellent in 46%, good in 27%, fair in 27% and poor in 0% of the patients with type C pelvic fractures. There were four postoperative infections. No sexual functional problem was reported. Neurologic problem like Lateral cutaneous nerve of thigh injury recovered completely in 2 patients and partially in 2 patients. There was no significant relation between functional outcome and the site of fracture (P greater than 0.005).

**Conclusions:**

Unstable pelvic ring fracture injuries should be managed surgically by rigid stabilization. It must be carried out as soon as the general condition of the patient permits, and even up to two weeks.

## Introduction

Pelvic ring disruptions are uncommon injuries occurring in 3 to 8.2% of all trauma patients.^1,2^ Pelvic fractures account for 1-3% of all skeletal fractures and 2% of orthopedic hospital admissions. The frequency of pelvic fractures occurs in a bimodal pattern, with peaks observed in persons aged 20-40 years and later in individuals older than 65 years.^3^ It is well established now that isolated posterior or combined posterior and anterior surgical fixation are required to achieve anatomical reduction and early ambulation in most, if not all, of unstable pelvic injuries.^4,5^ Different techniques of open reduction and internal fixation were introduced. The choice of a particular technique of internal fixation is influenced by the general condition, like concomitant injuries, nursing requirements of the patient and personal experience and bias of the surgeon. Each technique has inherent advantages as well as potential problems.^4,6,7,8^ Recently the evaluation of pelvic ring injuries and its management has moved from radiologic and early clinical results to functional results and quality of life-related issues.^1,10^ The most common high-energy mechanism of injury is a motor vehicle accident. Patients who sustain these injuries not only have skeletal injuries but also often have concomitant life-threatening injuries. Early death after these injuries is usually due to hemorrhage, multiple organs system failure, or sepsis (as high as 40 to 50%).^3^

However, associated skeletal injuries in patient with pelvic fracture might make overshadow impairment of pelvic fracture. Other considerations in the evaluation of pelvic fracture management are the type of pelvic injury, patients' age, the time of surgery, surgeon’s expertise of the treating and the presence of other system injuries. Therefore quantitative functional outcomes of pelvic fracture are influenced by different variables like associated injuries and the best method of decreasing these variables has not been established yet.^11^ This retrospective study was conducted to evaluate the radiological and functional results of surgical treatment of traumatic unstable pelvic injuries by internal fixation. The effect of associated skeletal injuries, patients' age, types of pelvic injuries and timing of internal fixation on the final outcomes were also studied separately.

## Methods

We evaluated patients with blunt pelvic fractures over a 7-year period. Inclusion required multiple pelvic fractures with vascular disruption and retroperitoneal hematoma, open book fracture with symphysis diastases, or sacroiliac disruption with vertical shear. (Between 2002 and 2008). Patients who were lost during follow up or died due to cause not related to their skeletal injuries were excluded from the study. There were 26 males and 12 females. The mean age was 37 ±10 years (range, 20-67). The causes of injuries were a road traffic accident in thirty seven patients and a fall from height in one patient. According to Tile classification, 27 patients had type-B fracture (Rotationally unstable) and11 patients had type-C fracture( Rotationally and vertically unstable). 

Anterior wall of pelvic ring distribution injury as follow as below; 7 cases with unilateral ramus fracture, 2 cases with bilateral ramus fracture and 28 cases with open fracture of symphysis pubis.

Posterior wall of pelvic distribution injury as follow as below; 12 cases with both sacroiliac dislocation and fracture, 2 cases with only sacroiliac dislocation, 13 cases with iliac fracture, and 4 cases with sacrum fracture who were associated with sacroiliac joint fracture. Patients with unstable pelvic ring fracture admitted in the Regional Trauma Center of Taleghani Hospital in Kermanshah (Iran), fractures of the pelvic ring after blunt trauma were identified from the trauma registry. Study inclusion required multiple pelvic ring fractures associated with vascular disruption and severe retroperitoneal hematoma, open book fracture with symphysis diastases, or sacroiliac disruption with vertical shear (all anterior-posterior compression fractures). All patients were evaluated by the trauma team in the resuscitation area. If initial assessment revealed on unstable pelvic fracture and the patient was hemodynamically labile, emergent vital signs stabilization was performed. Associated injuries have been followed by par clinic studies like CBC, blood pressure control, radiography and sonography studies.

After appropriate therapeutic and diagnostic measures were instituted, internal fixation was performed for twenty one patients during the first week of admission. Eleven patients had surgery within 48 hours after injury. Surgery was delayed for one week due to unstable general condition and some injury associated. In cases who had combined anterior and posterior pelvic wall fractures, the anterior pelvic wall stabilization was done first because of easier and most available for surgen and patient. Anterior stabilization was carried out by ilioinguinal approach; the patient has been placed on supine position, with hip flexed 20 to 30° to relax the psoas tendon. Incision starts from 2 fingerbreadths above the symphysis pubis, to anterior superior iliac spine, then two thirds along the iliac crest. Bone fixation has been done by five to ten hole plaque which is placed on the superior surface of the reduced symphysis. For posterior pelvic fracture stabilization, the patient is placed on prone position then by two, 4 hole plaque with 90 degree angle to each other applied on surface of sacroiliac region by Kocher-Langenbeck approach. The incision starts lateral to the anterior superior iliac spine, proceeds to the greater trochanter and then continues along the axis of the femur to almost the midpoint of the thigh. The advantages of this approach are femoral head lies in a reduced position, tendency for the femoral head to translate medially is eliminated and controlled traction is available by means of a fracture table while allowing flexion of the knee to relax the sciatic nerve.

For patient with acetabulum fracture, langenbeek approach has been performed. Acetabulum stabilization has been done with constractor plaque on displaced bone.

We didn’t use of iliofemoral approach for both columns fracture of pelvic ring, because of some disadvantages like approach to the anterior column isn’t as good as the ilio-inguinal approach. The longest postoperative recovery, the highest incidence of ectopic bone formation and the highest blood loss are disadvantages of ilio-inguinal approach.

After surgery, during the hospitalization, all patients received antibiotic against probably infection and low molecular weight heparin (Enoxaparin 20mg sc) for Prophylaxis against Deep Venous thrombosis. Prophylaxis indomethacin against heterotopic ossification was used and advised to all patients added their weight on affected pelvic belt step by step. 

The commonest associated injury was long bone fracture in lower extremity like femur and/or tibial fracture that was happened in 17(44.7%) patients. Other associated injures were urologic injury in 12 cases (31.5%), sustain bladder tear in 8 of 12 cases, urethral rupture in 3 of 12 cases and 1 of 12 had testis injury who were referred to urologist. Neurological injuries in 11 cases (29%) that 5 of 11 patients had lumbosacral plexus injury at the initial evaluation and 3 of 11 cases had combined motor and sensory deficits affecting the L4-L5, and S1 nerve roots and the remainder was an isolated sensory deficit of the S1 and S2 nerve roots.

There were 6 patients (15.7%) with intraabdominal hemorrhage which is approved by sonography. They underwent of urgent laparotomy that 2 of 6 patients had splenic rupture, two patients had colon rupture and 2 patients had spleen and liver injury.

Two patients (5.2%) had head injury who were referred to neurosurgeon, four patinets (10.5%) had upper extremity fractures (three patients with humerus and one patient with scapula fracture), two patients (5.2%) had L4 vertebral body fracture and one patient(5.2%) had pneumothorax. One patient had vaginal wall rupture. 

Patients were followed for at least seven years (range 16-48 months) after discharge from the hospital. At the last visit each patient was studied radiologically and functionally. Radiological outcome of fixation was determined through post-operative plain radiographs of the three standard views. The radiological result was graded by the maximum residual displacement in the posterior or anterior pelvic ring injuries as; excellent for 0 to 5mm, good for 6 to 10mm, fair for 11 to 15mm and poor for more than 15mm of displacement or established nonunion.^5^ The functional result was measured using the functional grading scale described by Majeed.^12^ Majeed functional scoring system consists of several questions in seven items. These items include pain, work, sitting, sexual intercourse, walking aids, gait and walking distance. They each score a number of points, which make up the total score ranging from 0 to 100. 

According to the total score patients were graded as excellent for 95, good for 85 to 94, fair for 70 to 84 and poor for less than 70 points.^5,9^ The advantage of this outcome scoring system is short, simple and can be used more practically in a clinical sitting. Also it includes sitting, which is a function often limited after pelvic injuries.

All follow up data was collected and patients were recalled for assessment of their radiological and functional outcome which was assessed for Majeed scoring system. The current job status was also recorded. Data were subjected to one way analysis of variance, where P<0.05 was considered significant.

## Results

Thirty eight patients were included with traumatic unstable pelvic fractures in our study. According to Tile’s classification of pelvic injuries,^13^ there were 27 patients with type B and 11 patients with type C pelvic fracture. 

All patients in our study managed by internal fixation for their anterior and posterior pelvic fractures. For 31 patients, anterior plaque fixation for symphyseal disruption were also performed by iliofemoral approach and for 31 patients with posterior pelvic fracture, posterior sacroiliac surface was fixed by two numbers four holes plaques with 90 degree angle to each other by Kocher-Lange beck approach. 

All patients discharged two to seven days after surgery except one who developed pulmonary embolism, in spite of receiving anticoagulant as a routine prophylactic measure , but finally the patient was treated successfully, after10 days discharged and total percent of complication was 34.2% (13 patients). The commonest postoperative complication was deep wound infection and lateral cutaneous nerve of thigh injury. There are no evidence of deep vein thrombosis. All patients were visited by an urologist, according to his report, there was no sexual and urinary dysfunctions. All fractures united except of one patient with type C pelvic fracture who had implant failure on pelvis and necessitated plate removal before healing of anterior pelvic disruption. Four patients who were successfully treated by oral antibiotics develop deep wound infection. Other post operative complications included in Table 1.

**Table 1:** Complication after surgery

Complication	Prevalence	Percent
Malposition	1	2.6
Deep wound infection	4	10.5
Lateral cutaneous nerve of thigh injury	4	10.5
Symphiseal fusion	1	2.6
Pelvic obliquity	1	5.2
Pulmonary thrombo-emboli	2	2.6
Device failure	1	2.6
Nonunion	1	2.6
Urinary tract infection	2	5.2

The average total hospital stay was 5 days (range 2-14). The average time to start mobilization after surgical stabilization was 9 days (range2-12) for patients who did not have serious skeletal injuries (12 patients) and 10.6 days (range 5-12) for patients who have skeletal injuries and walking problem(26 patients). 

The average follow-up time for all patients was 45.6 months (range 16-84). At the end of their follow-up all patients had union of their bony fractures except one. 

Radiological outcomes: The radiological result was graded by the maximum residual displacement in the posterior or anterior pelvic ring injuries as; excellent for 0 to 5mm, good for 6 to 10mm, fair for 11 to 15mm and poor for more than 15mm of displacement or established non union.^5^ Our results are shown in Table 2, which shows that radiologic outcomes were better for Type B fractures (p<0.003).

**Table 2:** Radiological outcomes after surgery

		Radiologic	outcome	
	Excellent	Good	Intermediate	weak
Type-B Fracture	73%(8)	18%(2)	0%(0)	9%(1)
Type-C Fracture	27%(7)	18%(5)	27%(7)	27%(7)
		p<0.003		

Clinical outcomes are shown in Table 3, which shows that clinical outcomes were better for Type B fractures (p<0.005). 

**Table 3:** Functional(clinical) outcomes after surgery

		Radiologic	outcome	
	Excellent	Good	Fair	weak
Type-B Fracture	66%(7)	15%(2)	11%(1)	8%(1)
Type-C Fracture	48%(13)	27%(7)	27%(7)	0%(0)
		P<0.005		

Thirteen of 27 patients with type B fractures were pain-free at the time of study as opposed to 3 of 11 patients with type C fractures. Three patients with type C fracture suffered from severe durable pain who couldn’t back to their work. Totally thirty five patients returned to their original job which shows that a durable pain is the most important factor to prevent returning to their job. 

Outcome evaluation: age and sex didn’t influence on radiological and clinical outcome but they were depended on the type of pelvic fractures (P<0.05).

Excellent radiological and clinical outcomes in Type-B was better than Type-C pelvic fractures. However, this could not be statistically proved due to small number of patients that were reviewed. All neurological injuries involving the lumbosacral plexus were fully recovered at the time of follow-up. Duration and severity of pain are more than in Type-C pelvic fractures. However the level of pain influenced their performance accordingly.

The difference between radiological and functional outcomes was found insignificant statistically (P>0.05). The effects of the differences in age of the patients, types of pelvic injuries and timing of internal fixation on final outcomes were all found statistically insignificant (P>0.05).

## Discussion

Pelvic fractures by high-energy traumas are severe lesions and relatively rare injuries, with significant mortality rate and a great number of associated lesions. Their incidence in trauma patients is quoted to range between 3 % and 8.2 % and instability occurs in 13 % to 17 % of cases.^14^ The commonest cause of a pelvic ring disruption is a Road Traffic Accident and that involved (37 patients)91% of our patients. Because of the large force that is required to disrupt the pelvis, pelvic fractures are indicative of high-energy transfer to the patient and therefore, often combined with other injuries.^14,15^

Early mortality in relation to fracture of the pelvis is due to associated injuries or catastrophic hemorrhage. About 10% of patients with hemodynamically unstable fractures of the pelvis will die.^16^ Late mortality in relation to an unstable fracture of the pelvis is most commonly because of sepsis.^17^ Other recently published evidence suggests that the severity of associated injuries is a better predictor of mortality than the presence of an unstable fracture of the pelvis pattern. 

Historically, pelvic ring injuries, depending on their severity had been treated by a variety of closed methods. Unstable pelvic injuries treated by these conventional measures often result in significant disability, moreover the mortality can reach 21.8%.^8,13,18^ There was a growing body of evidence that the application of an external skeletal frame will reduce venous and bony bleeding and improve tamponade by reducing and maintaining the pelvic volume to the extent that other interventions are rarely required.^6,7,13,19^ Recently biomechanical studies showed that external frame could not ensure sufficient stability to allow mobilization without the risk of redisplacement of the fragments particularly those with vertical instability. External fixators can be used temporarily in unstable injuries as part of emergency treatment to allow the patient to be placed with the trunk in the upright position to improve ventilation.^5,18,20,21,22^

Our results agree with other studies stating that anterior and/or posterior fixation could restore excellent stability and adequate consolidation of the unstable (Type-C) pelvic injuries with subsequent decrease in morbidity and mortality.^8,9,22,23^ The patients of this study had rapid improvement of their general condition with early discharge from hospital. They were mobilized relatively earlier without significant risk of re-displacement of the fragments (average9 days) in spite of associated other skeletal injuries .In cases where there were no other skeletal injuries who can walking earlier and average time to start mobilization was significantly (P<0.05) decreased to 2.5 days. 

It has been emphasized that surgical treatment should be carried out five to seven days post trauma when the patient general status allows.^8,22^

In the current study there was no statistical difference between the results of internal fixation of pelvic injuries carried out within two to three days after injury and who had delayed fixation about one week was due to unstable general condition (P>0.05). It is the author’s opinion to perform internal fixation for unstable pelvic injuries as soon as the general condition stabilized even up to two weeks after the injury. In our experience, fixation of the anterior ring disruption with five to ten holes plates is a simple procedure and provides satisfactory stabilization.

Iliosacral plaques fixation has recently become popular as it is minimally invasive and provides stable fixation using reasonably small implants. Biomechanically it is equal or superior to other techniques of internal fixation.^4,7,23,24,25^ This technique was used for all cases required posterior stabilization in this study and the results were satisfactory in 96% radiologically and in 82% functionally. The technique was very demanding, even in expert hands. Thorough understanding of the anatomy of posterior pelvis and their radiographic correlations are always necessary to reduce the complication rate.^4,7,26^ Different authors have reported that many factors associated with worse functional outcome including open fracture^24^ urological injuries include urethral, corpus cavernosa, bladder and bladder neck injuries,^27,32^ neurological injury,^28^ fractures requiring open reduction and internal fixation,^28,29^ residual posterior displacement^30^ and psychological problems.^31^ High-risk trauma patients have an increased risk of deep-vein thrombosis (DVT) and pulmonary embolism.^33^ Associated injuries occurred in most of our patients (36 out of 38 patients). The presence of associated injuries has certainly increased morbidity in our group of patients and negatively affected the functional outcome. The incidence of neurological injuries in this study was 29% which is comparable with that reported in the literature. Four patients with combined motor and sensory neurological deficits affecting the L4, L5, S1 nerve roots were fully recovered at the time of follow-up. One patients with isolated sensory deficit of S1 and S2 nerve roots were recovered at the time of follow-upa bout average 16 to 34 months following injury. The small number of patients in this study may be a reason for the most favorable potential for recovery of the neurological injury at the L4, L5, and S1 nerve roots, as compared with the literature.^34,12^ Open reduction and internal fixation of the unstable pelvic ring fractures provides the best stability of fixation as well as best clinical outcome.^23,35^ Our study in early rigid stabilization of both anterior and posterior pelvic ring injury, which is what was performed in our patients, has been suggested as a potential reason for favorable prognosis of these injuries.

Berner reported a rate of 16% unsatisfactory functional, and 17% unsatisfactory radiological result in a group of 42 patients treated non-operatively after combined disruptions of the pubic symphyses and the sacroiliac joint.^36^

After study current in treatment of a similar injury with open reduction and internal fixation, the rate of unsatisfactory functional rating was 8% in type C and 0% in type B. The rate of unsatisfactory radiological rating was 27% in type C and 8% in type B pelvic fracture. Three patients with type C pelvic fracture who had durable pain couldn’t back to original job.Our study showed that 35 patients returned to their original jobs. In the largest series of patients treated with open reduction and internal fixation of unstable posterior pelvic injuries, 67% returned to their former jobs without restrictions. ^29^

Although there is no statistical difference between radiological and functional results in the current study, the clinical figures agreed with the hypothesis that radiological outcome is usually better than the functional outcome. The functional results are often affected by the associated skeletal or extra skeletal injuries as well as other variables.^18,19,22^ Simultaneous effects of these variables on the final outcome make it impossible to study each effect separately. A huge number of cases are needed to accomplish this task by choosing patients with only one variable at a time.

## Conclusion

Pelvic fractures are challenging injuries to manage. Stabilization of vital parameters takes preference and significantly reduces mortality. Associated injuries are common and often have a substantial effect on the patient’s psychological status. Rehabilitation period is prolonged; however proper management yields a satisfactory outcome. Further analysis and studies including a larger number of patients are required to identify the prognostic factors for the late sequelae. This study should be a valid statistical analysis of outcomes in patients who treated surgically, by internal fixation. Early rigid stabilization of both anterior and posterior pelvic ring injury with open reduction, internal fixation, which is what was performed in our patients, has been suggested as a potential reason for favorable prognosis of these injuries.

## References

[1] Pohlemann T, Gansslen A, Schellwald O, Culemann U, Tscherne H. Outcome after pelvic ring injuries. Injury. 1996; 27 Suppl 2: B31-8. 8915200

[2] Gänsslen A, Pohlemann T, Paul C, Lobenhoffer P, Tscherne H. Epidemiology of pelvic ring injuries. Injury. 1996; 27 Suppl 1: S-A13-20. 8762338

[3] Korovessis P, Baikousis A, Stamatakis M, Katonis P (2000). Medium- and long-term results of open reduction and internal fixation for unstable pelvic ring fractures. Orthopedics.

[4] Xu R, Ebraheim NA, Robke J, Yeasting RA (1996). Radiologic evaluation of iliosacral screw placement. Spine (Phila Pa 1976)..

[5] Lindahl J, Hirvensalo E, Böstman O, Santavirta S (1999). Failure of reduction with an external fixator in the management of injuries of the pelvic ring. Long-term evaluation of 110 patients. J Bone Joint Surg Br.

[6] Wild JJ Jr, Hanson GW, Tullos HS (1982). Unstable fractures of the pelvis treated by external fixation. J Bone Joint Surg Am.

[7] Kregor PJ, Routt ML Jr. Unstable pelvic ring disruptions in unstable patients. Injury. 1999; 30 Suppl 2: B19-28. 10.1016/s0020-1383(99)90004-9 10562857

[8] Webb LX, Gristina AG, Wilson JR, Rhyne AL, Meredith JH, Hansen ST Jr (1988). Two-hole plate fixation for traumatic symphysis pubis diastasis. J Trauma.

[9] Van den Bosch EW, Van der Kleyn R, Hogervorst M, Van Vugt AB (1999). Functional outcome of internal fixation for pelvic ring fractures. J Trauma.

[10] Edeiken-Monroe BS, Browner BD, Jackson H (1989). The role of standard roentgenograms in the evaluation of instability of pelvic ring disruption. Clin Orthop Relat Res.

[11] Cole JD, Blum DA, Ansel LJ (1996). Outcome after fixation of unstable posterior pelvic ring injuries. Clin Orthop Relat Res.

[12] Majeed SA (1989). Grading the outcome of pelvic fractures. J Bone Joint Surg Br.

[13] Tile M (1988). Pelvic ring fractures: should they be fixed?. J Bone Joint Surg Br.

[14] Mucha P Jr, Farnell MB (1984). Analysis of pelvic fractures management. J Trauma.

[15] van Veen IH, van Leeuwen AA, van Popta T, van Luyt PA, Bode PJ, van Vugt AB (1995). Unstable pelvic fractures: a retrospective analysis. Injury.

[16] Dyer GS, Vrahas MS (2006). Review of the pathophysiology and acute management of haemorrhage in pelvic fracture. Injury.

[17] Kataoka Y, Minehara H, Shimada K, Nishimaki H, Soma K, Maekawa K (2009). Sepsis caused by peripelvic soft tissue infections in critically injured patients with multiple injuries and unstable pelvic fracture. J Trauma.

[18] Goldstein A, Phillips T, Sclafani SJ, Scalea T, Duncan A, Goldstein J (1986). Early open reduction and internal fixation of the disrupted pelvic ring. J Trauma.

[19] Kim WY, Hearn TC, Seleem O, Mahalingam E, Stephen D, Tile M (1999). Effect of pin location on stability of pelvic external fixation. Clin Orthop Relate Res.

[20] Gruen GS, Leit ME, Gruen RJ, Reitzman AB (1994). The acute management of haemodynamically unstable multiple trauma patients with pelvic ring fractures. J Trauma.

[21] Hirvensalo E, Lindahl J, Böstman O (1993). A new approach to the internal fixation of unstable pelvic fractures. Clin Orthop Relat Res.

[22] Tile M (1999). The management of unstable injuries of the pelvic ring. J Bone Joint Surg Br.

[23] Matta JM, Saucedo T (1989). Internal fixation of pelvic ring fractures. Clin Orthop Relat Res.

[24] Ebraheim NA, Coombs RJ, Hoeflinger MJ, Jackson WT (1993). A pitfall of radiologic evaluation of sacroiliac joint screw positioning. Orthopedics.

[25] Routt ML Jr, Simonian PT, Mills WJ (1997). Iliosacral screw fixation: early complications of the percutaneous technique. J Orthop Trauma.

[26] Brenneman FD, Katyal D, Boulanger BR, Tile M, Redelmeier DA (1997). Long-term outcomes in open pelvic fractures. J Trauma.

[27] Bjurlin MA, Fantus RJ, Mellett MM, Goble SM (2009). Genitourinary injuries in pelvic fracture morbidity and mortality using the National Trauma Data Bank. J Trauma.

[28] Suzuki T, Shindo M, Sorna K, Minehara H, Nakamura K, Uchino M (2007). Long-term functional outcome after unstable pelvic ring fracture. J Trauma.

[29] Tornetta P3rd, Dickson K, Matta JM (1996). Outcome of rotationally unstable pelvic ring injuries treated operatively. Clin Orthop Relat Res.

[30] Gruen GS, Leit ME, Gruen RJ, Garrison HG, Auble TE, Peitzman AB (1995). Functional outcome of patients with unstable pelvic ring fractures stabilized with open reduction and internal fixation. J Trauma.

[31] Kabak S, Halici M, Tuncil M, Avsarogullari L, Baktir A, Basturk M (2003). Functional outcome of open reduction and internal fixation for completely unstable pelvic ring fractures (type c): a report of 40 cases. J Orthop Trauma.

[32] Brandes S, Borrelli J Jr (2001). Pelvic fracture and associated urologic injuries. World J Surg.

[33] Sharma OP, Oswanski MF, Joseph RJ, Tonui P, Westrick L, Raj SS (2007). Venous thromboembolism in trauma patients. Am Surg.

[34] Helfet DL, Koval KJ, Hissa EA, Patterson S, DiPasquale T, Sanders R (1995). Intraoperative somatosensory evoked potential monitoring during acute pelvic fracture surgery. J Orthop Trauma.

[35] Tile M. Fractures of the pelvis and acetabulum. Baltimore: Williams and Wilkins, 1984.

[36] Berner W, Oestern HJ, Sorge J (1982). Ligamentous Pelvic ring injuries. Treatment and late results. Unfallheilkunde.

